# Serum Calprotectin Discriminates Subclinical Disease Activity from Ultrasound-Defined Remission in Patients with Rheumatoid Arthritis in Clinical Remission

**DOI:** 10.1371/journal.pone.0165498

**Published:** 2016-11-10

**Authors:** Jana Hurnakova, Hana Hulejova, Jakub Zavada, Martin Komarc, Petra Hanova, Martin Klein, Herman Mann, Olga Sleglova, Marta Olejarova, Sarka Forejtova, Olga Ruzickova, Jiri Vencovsky, Karel Pavelka, Ladislav Senolt

**Affiliations:** 1 Institute of Rheumatology, Prague, Czech Republic; 2 Rheumatology Department, First Faculty of Medicine, Charles University, Prague, Czech Republic; 3 Department of Methodology, Faculty of Physical Education and Sport, Charles University, Prague, Czech Republic; JAPAN

## Abstract

**Objective:**

Clinical remission in some patients with rheumatoid arthritis (RA) may be associated with ongoing synovial inflammation that is not always detectable on clinical examination or reflected by laboratory tests but can be visualized by musculoskeletal ultrasound. The goal of our study was to determine the levels of serum calprotectin, a major leukocyte protein, in patients with RA in clinical remission and to investigate the ability of serum calprotectin levels to distinguish patients in ultrasound-defined remission from those with residual ultrasound subclinical inflammation.

**Methods:**

Seventy RA patients in clinical remission underwent clinical and ultrasound examination. Ultrasound examination was performed according to the German US7 score. Ultrasound remission was defined as grey scale (GS) range 0–1 and power Doppler (PD) range 0. The levels of serum calprotectin and C-reactive protein (CRP) were determined. The discriminatory capacity of calprotectin and CRP in detecting residual ultrasound inflammation was assessed using ROC curves.

**Results:**

The total number of patients fulfilling the DAS28-ESR, DAS28-CRP, SDAI and CDAI remission criteria was 58, 67, 32 and 31, respectively. Residual synovial inflammation was found in 58–67% of the patients who fulfilled at least one set of clinical remission criteria. Calprotectin levels were significantly higher in patients with residual synovial inflammation than in those with ultrasound-defined remission (mean 2.5±1.3 vs. 1.7±0.8 μg/mL, p<0.005). Using ultrasound-defined remission criteria, calprotectin had an AUC of 0.692, p<0.05 using DAS28-ESR remission criteria and an AUC of 0.712, p<0.005 using DAS28-CRP remission criteria. Calprotectin correctly distinguished ultrasound remission from subclinical activity in 70% of patients. CRP (AUC DAS28-ESR = 0.494, p = NS; AUC DAS28-CRP = 0.498, p = NS) had lower and insignificant discriminatory capacity.

**Conclusion:**

The present study demonstrates the potential of calprotectin to distinguish RA patients in both clinical and ultrasound-defined remission from patients in clinical remission but with residual subclinical disease activity.

## Introduction

Rheumatoid arthritis (RA) is a chronic inflammatory autoimmune disease characterised by persistent symmetric synovitis, the development of joint deformities and bone destruction. The introduction of new drugs and strategies, more accurate assessments of joint inflammation and tighter control of disease activity have markedly increased rates of clinical remission [1, 2]. Remission, which is ideally regarded as an absence of inflammation, synonymous with no clinical symptoms or signs, should result in optimal structural and functional outcomes [3]. Nevertheless, it has been demonstrated that some patients who are in clinical remission may experience functional worsening and radiographic progression [4–6]. Recent data clearly showed that the use of sensitive imaging techniques (e.g., magnetic resonance imaging or ultrasonography) permits the detection of residual subclinical disease activity despite clinical remission, which may explain continuing structural progression [7, 8]. Some patients in clinical remission therefore do not have a lack of joint inflammation; rather, they have low disease activity that is not easily detectable by clinical examination or conventional laboratory tests [9]. Thus, more accurate methods for evaluating remission status are needed.

Musculoskeletal ultrasonography is a modern imaging method that enables direct visualization of joint structures [10]. Furthermore, it is more successful in detecting synovitis than clinical examination and may be able to quantify the extent of inflammation and to distinguish active inflammatory processes from chronic synovial hypertrophy [11–14]. In addition, recent data suggested that ultrasonography reveals subclinical disease activity [15, 16] and that even small amounts of imaging-detected synovial inflammation predict relapse [17] and radiographic progression despite clinical remission [8, 18].

Calprotectin (also known as S100A8/A9[19] and MRP8/MRP14[[20]) is a major leukocyte cytosolic protein released during inflammatory processes, predominantly from locally activated leukocytes at the sites of joint inflammation, and thus directly reflects joint inflammatory activity rather than systemic inflammation [21]. High concentrations of this protein were found in the synovial fluid of active arthritis joints [22]. Moreover, several investigators have reported increased serum levels of calprotectin in RA patients and have demonstrated calprotectin’s correlation with disease activity [23, 24] and its decrease in response to effective treatment [25]. Recently, a significant correlation between calprotectin and ultrasound-determined synovial inflammation was demonstrated in RA patients [26]. We have shown that calprotectin might be a more sensitive tool for assessing joint inflammation and a better predictor of ultrasound synovitis than conventionally used C-reactive protein (CRP) [27]. Thus, we suggested that calprotectin and musculoskeletal ultrasonography might provide more reliable tools for evaluating joint inflammatory activity in RA patients. In line with our hypothesis, Inciarte-Mundo et al [28] have recently demonstrated that calprotectin and TNF trough serum levels may help to identify power Doppler ultrasound synovitis in biologic-treated patients with RA and psoriatic arthritis in remission or low disease activity.

Until now, no studies have focused only on RA patients in clinical remission and the accuracy of calprotectin in distinguishing patients in ultrasound remission from those with residual ongoing inflammation detected by ultrasonography. Therefore, the aims of the present study were (1) to analyse serum levels of calprotectin in RA patients in clinical remission according to a several composite indices and (2) to investigate the accuracy of serum calprotectin and CRP levels in distinguishing patients in ultrasound remission from those in clinical remission with residual ultrasound disease activity.

## Patients and Methods

### Patients

A total of 70 patients whose RA was in clinical remission according to the treating rheumatologist in the outpatient department of the Institute of Rheumatology in Prague were enrolled into this study. Diagnosis of RA was based on fulfilment of the ACR/EULAR 2010 classification criteria [29]. All patients were older than 18 years. The study was performed according to the guidelines of the Declaration of Helsinki and was approved by the local ethics committee of the Institute of Rheumatology in Prague. All included patients gave their written informed consent before entry into the study. One trained nurse performed the clinical assessment of tender and swollen joints. Disease activity was assessed by the DAS28 score, which includes the swollen joint count (SJC) and tender joint count (TJC), the erythrocyte sedimentation rate (ESR) or C-reactive protein (CRP) and the patient's and physician's global assessment of activity on the visual analogue scale (VAS), the Simplified Disease Activity Index (SDAI) and the Clinical Disease Activity Index (CDAI) [30–32]. Clinical remission criteria were defined as DAS28<2.6, SDAI≤3.3 and CDAI≤2.8.

### Laboratory assessment

Fasting blood samples were obtained on the day of the clinical and ultrasound examinations. Blood samples were centrifuged and stored at –80°C until the analysis. Calprotectin was measured using a commercially available enzyme-linked immunosorbent assay (ELISA) according to the manufacturer’s protocol (Bühlmann Laboratories AG, Schőnenbuch, Switzerland). The inter-assay and intra-assay reliability of the S100A8/9 assays were 5.8% and 4.3%, respectively, and the detection limits were 0.4 μg/mL. ESR was measured on BD-15^™^ instrument (BD, New Jersey, USA). CRP level was measured using turbidimetry (Beckman (Beckman Coulter, California, USA). Anti-cyclic citrullinated peptide (anti-CCP) antibodies and rheumatoid factors were analysed using standard ELISA kits (Test Line s.r.o., Brno, Czech Republic).

### Ultrasound imaging

The ultrasound examinations were performed with Esaote Mylab 60 equipment (Esaote S.p.A., Genova, Italy) using a linear transducer with a 12–18 MHz frequency. Each patient was examined according to the German US7 score in the following seven joint areas: wrist, second and third metacarpophalangeal joints, second and third proximal interphalangeal joints, and second and fifth metatarsophalangeal joints of the clinically more affected hand and foot [33]. We used a modification of the original German US7 [34]. In contrast to the original US7, which assesses synovitis in MCP and PIP joints using grey scale (GS) imaging only from the palmar view, we assessed synovitis in this area using GS imaging from both the palmar and dorsal views. Synovitis assessed using GS imaging was scored semiquantitatively (0 = none, 1 = mild, 2 = moderate, 3 = severe synovitis), as follows: grade 1 = a small hypoechoic/anechoic line beneath the joint capsule; grade 2 = the joint capsule elevated parallel to the joint area; and grade 3 = a strong distension of the joint capsule. Synovitis was classified semiquantitatively by power Doppler (PD) ultrasound as follows: grade 0 = no intraarticular colour signal; grade 1 = up to 3 colour signals or 2 single signals and 1 confluent signal in the intraarticular area; grade 2 = greater than grade 1 to < 50% of the intraarticular area filled with colour signals; and grade 3 = ≥ 50% of the intraarticular area filled with colour signals. An overall GS and PD signal score was calculated as the sum of GS synovitis and PD synovitis. The GS synovitis scores range from 0–39, and the PD synovitis scores range from 0–39. The tenosynovitis sum score was not assessed in this study. Ultrasound remission was defined as GS 0–1 and PD 0. The ultrasonographers were blinded to each patient’s clinical examination and laboratory findings. The inter- and intra-observer reliability was moderate to very good, as shown in a recent study [27].

### Statistical analysis

The data are presented as the mean and standard deviation (SD) unless stated otherwise. The basic descriptive statistics (the mean, median, standard deviation, skewness and kurtosis) were computed for all of the variables, which were subsequently tested for normal distribution using the Kolmogorov-Smirnov test. A t-test was used for normally distributed variables and the Mann—Whitney test as a nonparametric alternative to analyse the differences between the two groups. Receiver operating curves (ROC) and the area under the curve (AUC) were used to compare the discriminatory capacities of calprotectin and CRP to identify ultrasound remission. The best cut-offs in terms of sensitivity and specificity were identified, and the percentage of correct classifications were computed. Multivariate regression analysis was performed with calprotectin, age, sex, disease duration as well as RF and ACPA status as the independent variables in predicting residual ultrasound activity. Statistical significance was set as P values less than 0.05. The statistical analysis was carried out using SPSS version 23 statistical software (SPSS, Inc., Chicago, IL, USA).

## Results

### Baseline patients characteristics

All patient characteristics are shown in Table 1. The study population was predominantly of female, and the mean (SD) age was 56.7±13.4 years. The mean (SD) symptom duration was 5.6±5.3 years. RF and anti-CCP positivity were found in 60% (42/70) and 63% (44/70) of RA patients, respectively. At the time of examination, 86% (60/70) of patients were being treated with conventional synthetic disease-modifying anti-rheumatic drugs. Fifty-two patients were being treated with methotrexate (mean dose: 13.8 mg/week; range: 5 to 20 mg/week), three patients were being treated with sulfasalazine (all with a daily dose of 2 g) and five patients were being treated with leflunomide (mean daily dose: 14 mg; range: 10 to 20 mg). One patient was being treated concomitantly with golimumab (mean monthly dose: 50 mg) and leflunomide (mean daily dose: 20 mg). Nineteen patients were receiving glucocorticoids (mean daily dose: 4.4 mg of prednisolone or its equivalent; range: 0.5 to 5 mg). Seven patients in stable remission were not receiving treatment.

**Table 1:** Baseline characteristics of the patients with rheumatoid arthritis.

Characteristics	RA patients (n = 70)
Female (%)	51 73%
Age (years)	56.7 ± 13.4
RF positivity, n (%)	42 60%
Anti-CCP positivity, n (%)	44 63%
Calprotectin, μg/mL	2.1 ± 1.2
CRP, mg/L	3.8 ± 19
ESR, mm/1^st^ hour	13 ± 19
DAS28-ESR remission (No.) (%)	59/70 84%
DAS28-CRP remission (No.) (%)	67/70 96%
SDAI remission (No.) (%)	32/70 45%
CDAI remission (No.) (%)	31/70 44%

The values are the mean ± SD (range), unless stated otherwise.

Anti-CCP, anticyclic citrullinated peptide antibody; CDAI, Clinical Disease Activity Index; CRP, C-reactive protein; DAS28-CRP, Disease Activity Score for 28 joints with C-reactive protein; DAS28-ESR, Disease Activity Score for 28 joints with erythrocyte sedimentation rate; ESR, erythrocyte sedimentation rate; F, female; RA, rheumatoid arthritis; RF, rheumatoid factor; SDAI, Simplified Disease Activity Index

### Fulfilment of established criteria for remission

Of all the RA patients who were judged by their treating physicians to be in remission, 84% (59/70) fulfilled the DAS28-ESR remission criteria and 96% (67/70) were in remission according to the DAS28-CRP. However, only 45% (32/70) satisfied the SDAI remission criteria, and only 44% (31/70) satisfied the CDAI remission criteria, confirming the more stringent character of the SDAI/CDAI definition of remission (Table 2).

**Table 2:** Laboratory and ultrasound variables in patients who achieved clinical and ultrasound remission.

	Ultrasound remission		
	yes	no	p
DAS28-ESR remission (N = 59)			
Calprotectin, mean ± SD, μg/mL	1.7 ± 0.8	2.5 ± 1.3	0,016
Calprotectin, median (range), μg/mL	1.5 (0.4–3.4)	2.1 (0.6–6.9)	
CRP, mean ± SD, mg/L	3.6 ± 11.úno	3.5 ± 14.3	0,97
CRP, median (range), mg/L	2.8 (0.18–16)	2 (0.2–18)	
GS syn score (0–39) (mean)	0.45 ± 0.5	3.46 ± 2.2	0,001
PD syn score (0–39) (mean)	0 ± 0	1.5 ± 1	0,001
No of patients (%)	22 (37%)	37 (63%)	
DAS28-CRP remission (N = 67)			
Calprotectin, mean ± SD, μg/mL	1.6 ± 0.8	2.5 ± 1.3	0,007
Calprotectin, median (range), μg/mL	1.4 (0.4–3.4)	2.1 (0.6–6.9)	
CRP, mean ± SD, mg/L	3.57 ± 03.čvc	4 ± 5.6	0,714
CRP, median (range), mg/L	2.8 (0.18–16)	2.3 (0.2–24)	
GS syn score (0–39) (mean)	0.5 ± 0.51	3.73 ± 2,2	0,001
PD syn score (0–39) (mean)	0 ± 0	1.6 ± 1.5	0,001
No of patients (%)	22 (33%)	45 (67%)	
CDAI remission (N = 31)			
Calprotectin, mean ± SD, μg/mL	1.7 ± 0.9	2.6 ± 1,3	0,076
Calprotectin, median (range), μg/mL	1.7 (0.4–3.4)	2.3 (0.7–6.9)	
CRP, mean± SD, mg/L	3.2 ± 02.dub	4.8 ± 7,3	0,474
CRP, median (range), mg/L	2.9 (0.8–9)	1.9 (0.2–24)	
GS syn score (0–39) (mean)	0.5 ± 0.5	3.2 ± 1,8	0,001
PD syn score (0–39) (mean)	0 ± 0	1.3 ± 1	0,001
No of patients (%)	13 (42%)	18 (58%)	
SDAI remission (N = 32)			
Calprotectin, mean ± SD, μg/mL	1.8 ± 0.9	2.4 ± 1,4	0,166
Calprotectin, median (range), μg/mL	1.7 (0.4–3.4)	2.1 (0.7–6.9)	
CRP, mean± SD, mg/L	3.2 ± 2.36	2.9 ± 2,9	0,756
CRP, median (range), mg/L	2.9 (0.8–9.3)	1.7 (0.2–10)	
GS syn score (0–39) (mean)	0.5 ± 0.52	3.1 ± 1,6	0,001
PD syn score (0–39) (mean)	0 ± 0	1.4 ± 1	0,001
No of patients (%)	13 (41%)	19 (59%)	

CDAI; Clinical Disease Activity Index; CRP, C-reactive protein; DAS28-CRP, Disease Activity Score for 28 joints with C reactive protein; DAS28-ESR, Disease Activity Score for 28 joints with erythrocyte sedimentation rate; ESR, erythrocyte sedimentation rate; GS syn score, Grey Scale synovitis score; PD syn score; Power Doppler synovitis score; SDAI, Simplified Disease Activity Index

### Ultrasonography findings

In the entire cohort, 76% (53/70) of the patients showed GS evidence of synovial hypertrophy and 51% (36/70) had a positive PD signal. For these cases, the GS sum score ranged from 0 to 11 (median value 2), and the PD count ranged from 0 to 6 (median value 0). Complete remission according to the imaging results (GS 0/1 and PD 0) was found in 33% (23/70) of patients.

**Fig 1:**
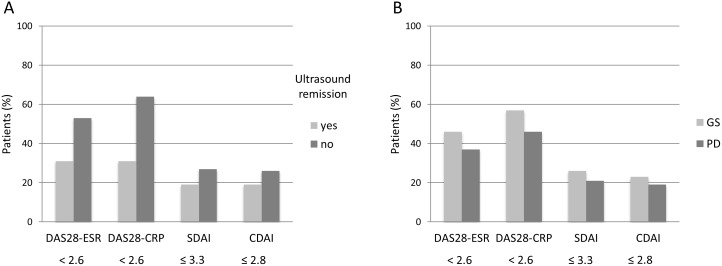
Presence of ultrasound findings in RA patients in clinical remission. (A) Percentage of patients fulfilling the ultrasound remission criteria. (B) Presence of positive GS and PD ultrasound findings.

In patients fulfilling the DAS28-ESR remission criteria, 54% (32/59) had positive GS findings, and 44% (26/59) showed increased vascularity on the PD ultrasound. Complete ultrasound remission was found in 37% (22/59) of patients (Table 2) (Fig 1). In the group of patients satisfying the DAS28-CRP remission criteria, 58% (39/67) had positive GS results, and 51% (34/67) showed increased vascularity on the PD ultrasound. Complete ultrasound remission was found in 33% (22/67) of patients. Among patients fulfilling the SDAI-based remission criteria, GS synovial hypertrophy was observed in 53% (17/32) of patients, and PD synovial hypertrophy was observed in 31% (10/32) of patients. Ultrasound remission was observed in 40% (13/32) of patients. Similarly, in patients in remission according to the CDAI criteria, synovial hypertrophy was detected via GS ultrasound in 52% (16/31) of patients, and via PD ultrasound in 42% (13/31) of patients. Ultrasound remission was observed in 42% (13/31) of patients.

### Calprotectin and CRP levels according to clinical and ultrasound remission

**Fig 2:**
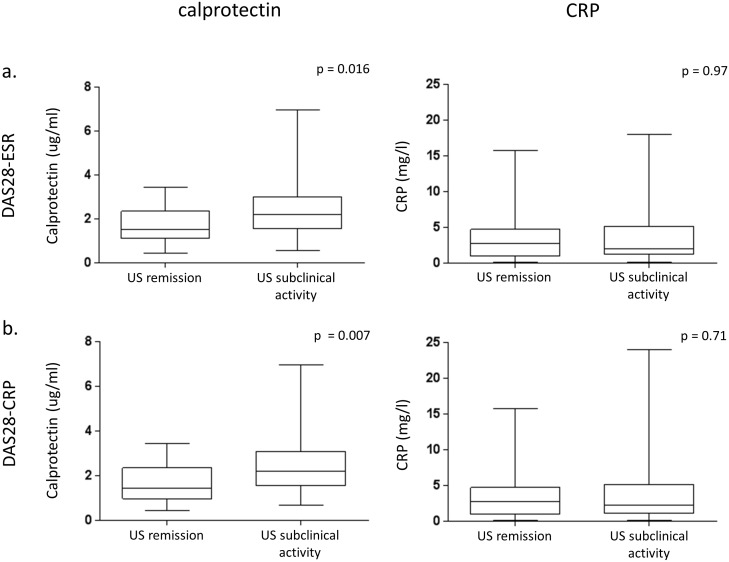
Box-plots showing levels of serum calprotectin and CRP in patients who achieved clinical remission according to a) DAS28-ESR and b) DAS28-CRP.

Of the patients who achieved clinical remission according to the DAS28-ESR and DAS28-CRP criteria, serum calprotectin levels were significantly lower in those who fulfilled ultrasound remission criteria than in those who had residual ultrasound disease activity (1.7±0.8 vs. 2.5±1.3 μg/mL, p = 0.016 and 1.7±0.8 vs. 2.5±1.3 μg/mL, p = 0.007, respectively). However, there was no difference in CRP level between these two groups (mean 3.6±11.2 vs. 3.5±14.3 mg/L, p = 0.97 and 3.6±3.7 vs. 4±5.6 mg/L, p = 0.7, respectively) (Fig 2). Using the SDAI and CDAI remission criteria, there lower levels of serum calprotectin were found in patients in ultrasound remission than in those with subclinical ultrasound disease activity (1.8±0.9 vs. 2.4±1.4 μg/mL, p = 0.17 and 1.7±0.9 vs. 2.6±1.3 μg/mL, p = 0.07, respectively). The levels of CRP did not differ between these two groups (3.2±2.4 vs. 2.9±2.9 mg/L, p = 0.76 and 3.2±2.4 vs. 4.8±7.3 mg/L, p = 0.47, respectively).

### Ability of calprotectin and CRP to identify patients in clinical and ultrasound remission

The ROC analysis with ultrasound remission as the reference variable showed an AUC for DAS28-ESR of 0.692, p = 0.015 (95% CI 0.552 to 0.832), with a cut-off calprotectin level of 1.7 μg/mL (sensitivity 72%, specificity 64%). The DAS28-CRP AUC for calprotectin levels was 0.712, p<0.005 (95% CI 0.58 to 0.845), with a cut-off calprotectin level of 1.7 μg/mL (sensitivity 71%, specificity 68%). Thus, calprotectin correctly distinguished patients in ultrasound remission from those with subclinical ultrasound activity in 70% of the cases.

**Fig 3:**
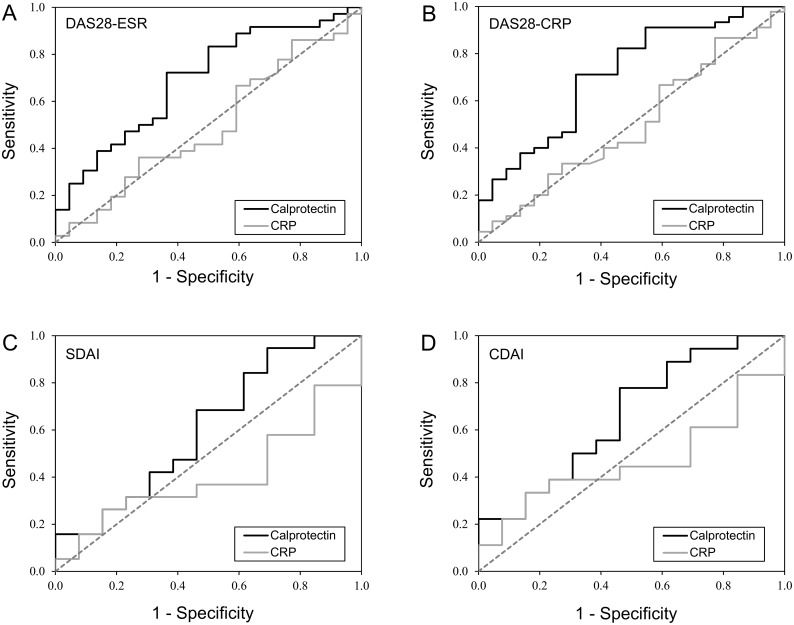
Receiver-operator characteristic curves of serum calprotectin and CRP for distinguishing patients with ultrasound remission from those with subclinical ultrasound activity. All the patients achieved clinical remission according to a) DAS28-ESR, b) DAS28-CRP, c) SDAI and d) CDAI criteria.

CRP (AUC DAS28-ESR = 0.494, p = 0.943; AUC DAS28-CRP = 0.498, p = 0.979) had an insignificant discriminatory capacity that was lower than calprotectin (Fig 3).

When using the more stringent SDAI/CDAI remission criteria, the AUC for calprotectin levels was higher (AUC SDAI = 0.607, 95% CI 0.401 to 0.813, p = 0.309; AUC CDAI = 0.658, 95% CI 0.460 to 0.856, p = 0.139) than for CRP (AUC SDAI 0.405, p = 0.367; AUC CDAI = 0.466, p = 0.749). However, its discriminatory capacity was statistically not significant. Calprotectin correctly distinguished 69% and 68% of patients in ultrasound remission from those with residual activity who also fulfilled the SDAI or CDAI remission criteria, respectively. In contrast, CRP correctly identified only 50% and 55% of patients who met the SDAI and CDAI criteria, respectively (Fig 3).

### Associations between calprotectin, age, sex, disease duration, use of glucocorticoids and methotrexate, autoantibodies status and residual US synovitis

In a multivariate regression analysis, calprotectin (OR = 3.2, 95%CI 1.3–7.8, p = 0.011) along with age (OR = 1.02, 95%CI 1.02–1.15, p = 0.011) but not sex, disease duration, use of glucocorticoids nor methotrexate, RF positivity nor ACPA status contributed significantly to predict ultrasound residual activity as the dependent variable (Table 3).

**Table 3:** Multiple regression analyses predicting residual ultrasound activity in rheumatoid arthritis patients in clinical remission.

Model		β	OR	95% Confidence Interval		p
				Lower Bound	Upper Bound	
	Calprotectin	1,16	3,189	1,31	7,764	0,011*
	Age	0,078	1,081	1,018	1,148	0,011*
	Disease duration	-0,002	0,998	0,986	1,01	0,711
	Sex	-1,428	0,24	0,044	1,319	0,101
	Glucocorticoids, yes or no	-0,313	0,731	0,141	3,801	0,71
	Methotrexate, yes or no	-1,484	0,227	0,033	1,54	0,129
	RF positivity	0,165	1,18	0,244	5,713	0,837
	ACPA positivity	0,558	1,747	0,369	8,269	0,482
	Constant	-4,431	0,012			0,025

Dependent Variables: US residual activity

* Correlation is significant at the p<0.05 level

Abbreviations: RF, rheumatoid factor; ACPA, anti-citrullinated protein antibody

## Discussion

In the present study we have shown a significant difference in calprotectin, but not CRP levels, between RA patients in clinical remission with residual disease activity detected by ultrasonography and those fulfilling ultrasound remission criteria. In addition, we have demonstrated that calprotectin is able to discriminate RA patients in ultrasound remission from patients with subclinical ongoing synovial inflammation who fulfilled other clinical remission criteria.

Remission is the ultimate goal of modern treatment strategies [35]. However, in some patients in clinical remission, low-grade inflammatory activity may persist but remain undetectable by routine examinations. In our study, we have focused on modern imaging techniques and a serum biomarker that may help identify patients in deep remission. These tools are useful for making decisions regarding drug withdrawal or tapering to minimize the risk of short-term relapse or for identifying patients with a risk of structural progression despite an apparent clinical response.

Indeed, the presence of even minimal PD signals in RA patients in clinical remission has been shown to be the strongest predictor of short-term relapse [17]. The association between PD and structural deterioration has also been demonstrated [8]. Importantly, it has been shown that negative PD has high negative predictive value of relapse and is associated with stable remission, supporting the concept that a negative PD finding is a more accurate remission criterion [17]. Conversely, minimal synovial changes detected by GS were also found in healthy subjects, suggesting that abnormalities of a low grade (grade 1) should not be considered pathological [36, 37]. Therefore, ultrasound remission criteria were defined as GS 0–1 and PD 0 in this study.

In our study sample, using the above-mentioned ultrasound remission criteria, we found that the majority of patients who achieved remission status according to the DAS28-ESR, DAS28-CRP or SDAI, CDAI criteria continue to have residual synovial inflammation when assessed with objective imaging techniques, which was in agreement with several previously published reports [7–9, 38]. Among the patients who met the DAS28 remission criteria (DAS28<2.6), only 33–37% patients also fulfilled the ultrasound remission criteria, and 63–67% patients experienced residual ultrasound-detectable synovial inflammation. When SDAI or CDAI remission criteria were applied, the ultrasound remission rate was slightly higher (41–42%), which reflects the greater strictness of the SDAI/CDAI remission criteria. Still, ultrasound features indicating persistent inflammation were found in a considerable number of patients (58–59%). Thus, we believe that ultrasonography may have an additional role in more precise evaluation of real disease activity, revealing persistent joint activity and confirming true remission—especially when clinical findings or traditional serum markers of inflammation have normalized.

However, modern imaging techniques may not be available in all rheumatology practices. More importantly, the results still depend on the skill of the ultrasonographer, which impedes the widespread use of this tool in the daily management of RA. Thus, circulating biomarkers of joint inflammation that can be comfortably measured along with other regularly provided blood tests would be remarkably useful for clinical practice.

Recently, we demonstrated an association between serum calprotectin and clinical and laboratory markers of disease activity and, more relevantly, with ultrasound synovitis, which is generally considered to be more precise in the evaluation of joint inflammation [27]. Moreover, we found that serum calprotectin might be a better predictor of ultrasound-determined synovitis than CRP [27]. Therefore, we investigated if serum calprotectin could identify patients in deep remission as determined by musculoskeletal ultrasonography. Regardless of the clinical remission criteria used in this study, we found significantly lower levels of serum calprotectin in patients with no objective ultrasonographic signs of synovial inflammation than in patients with subclinical ongoing synovitis. Whether evaluated using the DAS28 remission criteria or the more stringent SDAI or CDAI remission criteria, serum calprotectin differed significantly between patients with and without residual PD synovitis. However, there was no significant difference in CRP level between these two groups.

The hypothesis of our study was based on the suggestion that calprotectin is more sensitive to joint inflammation than CRP, which is primarily of hepatic origin. Calprotectin is predominantly produced locally at the sites of joint inflammation and therefore directly reflects the amount of activated macrophages and the extent of local synovial inflammation rather than a systemic response. Confirming this finding, Ramirez et al. [39] recently demonstrated that macrophage infiltration is comparable between patients with asymptomatic ultrasound-defined synovitis and clinically active arthritis. Therefore, we suggest that macrophage infiltration in the synovial tissue of RA patients who are in clinical remission but have residual PD synovitis represents a major source of calprotectin that may easily be diffused from inflamed joints into the blood circulation.

We showed that, using the DAS28 remission criteria, calprotectin, but not CRP, had a significant capacity to distinguish RA patients in ultrasound-defined remission from those with residual synovial inflammation. When we used SDAI/CDAI remission criteria, the AUC was higher for calprotectin than for CRP, although this difference was not statistically significant. This difference may have been caused by a small number of patients achieving the stricter SDAI/CDAI remission criteria. However, it is important to note that CRP forms part of the DAS-CRP and SDAI constructs, and patients satisfying the remission criteria defined by these indexes are inherently those in whom CRP failed to discriminate US-detected subclinical inflammation. Our results are in line with those published recently by Inciarte-Mundo et al. who had investigated levels of serum calprotectin and its ability to identify PD synovitis in patients with RA and psoriatic arthritis in clinical remission and low disease activity treated by antiTNFα agents [28]. In contrast to above mentioned study, we have focused here more strictly only on patients in clinical remission. Another difference is that, except of one patient treated with golimumab, our study consisted of biologic naïve RA patients in contrast to previous report.

In the present study, we determined specific cut-off levels for identifying patients in ultrasound-defined remission. The mean level of calprotectin in our cohort was 2.1 μg/mL, and the best cut-off level proposed for calprotectin to distinguish patients with and without deep ultrasound remission was 1.7 μg/mL, which was in line to those found by Inciarte-Mundo et al [28]. Using this cut-off, calprotectin levels correctly distinguished ultrasound remission from subclinical activity in 70% of RA patients. Based on our findings, serum calprotectin may distinguish patients with stable remission from those with residual synovial inflammation.

Our study has several limitations. The first limitation is the relatively small sample size of the subgroups. Second, the cut-off point of calprotectin associated with ultrasound remission or residual activity in RA should be interpreted with caution and merits further investigation in studies with larger sample sizes to establish its clinical significance. Further studies exploring the value of calprotectin in determining deep remission will be needed to confirm the findings in independent cohorts.

## Conclusions

This study shows the potential ability of serum calprotectin to discriminate ultrasound-defined remission from subclinical disease activity in RA patients who have achieved clinical remission. Overall, this study suggests that calprotectin might represent a valuable serological biomarker for confirming deep remission in RA patients.
